# Multi-omics revolution to promote plant breeding efficiency

**DOI:** 10.3389/fpls.2022.1062952

**Published:** 2022-12-08

**Authors:** Umer Mahmood, Xiaodong Li, Yonghai Fan, Wei Chang, Yue Niu, Jiana Li, Cunmin Qu, Kun Lu

**Affiliations:** ^1^ Integrative Science Center of Germplasm Creation in Western China (Chongqing) Science City and Southwest University, College of Agronomy and Biotechnology, Southwest University, Chongqing, China; ^2^ Academy of Agricultural Sciences, Southwest University, Chongqing, China; ^3^ Engineering Research Center of South Upland Agriculture, Ministry of Education, Chongqing, China

**Keywords:** multi-omics, breeding, genomics, proteomics, transcriptomics, metabolomics

## Abstract

Crop production is the primary goal of agricultural activities, which is always taken into consideration. However, global agricultural systems are coming under increasing pressure from the rising food demand of the rapidly growing world population and changing climate. To address these issues, improving high-yield and climate-resilient related-traits in crop breeding is an effective strategy. In recent years, advances in omics techniques, including genomics, transcriptomics, proteomics, and metabolomics, paved the way for accelerating plant/crop breeding to cope with the changing climate and enhance food production. Optimized omics and phenotypic plasticity platform integration, exploited by evolving machine learning algorithms will aid in the development of biological interpretations for complex crop traits. The precise and progressive assembly of desire alleles using precise genome editing approaches and enhanced breeding strategies would enable future crops to excel in combating the changing climates. Furthermore, plant breeding and genetic engineering ensures an exclusive approach to developing nutrient sufficient and climate-resilient crops, the productivity of which can sustainably and adequately meet the world’s food, nutrition, and energy needs. This review provides an overview of how the integration of omics approaches could be exploited to select crop varieties with desired traits.

## 1 Introduction

Since the origin of agriculture, food security has been one of the utmost precedence contemplations. In turn, plant breeding is one of the oldest agricultural activities that developed along with human civilization and a foremost method to meet the upsurged food demand. Humans have been cultivating and selecting crops that would serve their taste, nutritional values, high yield, and resistance to biotic and abiotic environments (Dossa et al., 2017). After the discovery of Mendel’s laws (1866) plant breeding enter a new era (Mendal, 1866), afterward pedigree breeding was developed based on the hybridization principle. The discovery of DNA structure (1863-1865) revolutionized plant breeding in the molecular era (Watson and Crick, 1953); thereupon, new breeding techniques were introduced such as marker-assisted selection (MAS) and the genetically modified (GM) approach (Shen et al., 2022). These discoveries shifted plant breeding from utter phenotype-selection to a combination of genotype and phenotype selection. Based on technical innovations plant breeding has been divided into four prominent categories; ensuant selection by farmers, statistical and experimental approach to improve selection, the convergence of genetic and genomic data, and the currently progressing era of optimal and precise design breeding (Shen et al., 2022).

Presently, it is a big challenge to feed the exponentially growing world population in changing climate for agriculture, particularly due to the diminution of fertile land with the incessant conversion of fertile land to semi-arid and nutrient deficient areas alongside salinity and waterlogging. The crop production is already under risk due to climate change, mainly staple food crops such as rice, maize, and wheat (Farooq et al., 2022; Syed et al., 2022). Crop productivity will be significantly impacted in the next decades by climate factors such as extreme temperature stresses, nutrient toxicity, or deficiency, changes in precipitation intensity and frequency, and other climate change-driven problems including salinity, waterlogging, drought, and land degradation (Teshome et al., 2020; Mahmood et al., 2021; Raza et al., 2021a). In addition to the detrimental effects of abiotic stress on plants, climate change has also exacerbated the impact of biotic constraints (insect and fungus), which significantly reduce the crop yield (Vaughan et al., 2018). These environmental constraints are the primary cause of the declining food productivity, which directly impacts economies worldwide. The basis for the sustained production of new varieties to address current and upcoming issues is the genetic diversity of crop plants.

Hence, an expeditious method of introducing elite climate-smart cultivars with desired traits (stress tolerance, yield and nutrition) is requisite. Plant breeding has always been pivotal for developing agriculture to produce sufficient food for a growing population. Its efficiency is tremendously improved by the technological innovations through OMICS approaches (genomics, transcriptomics, proteomics, metabolomics, and phenomics) and ensued greater food supply to meet the ever-increasing demand (Muthamilarasan et al., 2019; Yang et al., 2021). Multi-omics studies of plants have played a crucial role in interpreting metabolic pathways and their molecular regulators that control key traits and the growth processes of multiple plant species (Razzaq et al., 2019). Recent advances in next-generation sequencing (NGS) technologies have managed high throughput and swift data generation for OMICS experiments (Großkinsky et al., 2017; Schmidt et al., 2020), which has improved the accuracy, sensitivity, and detection throughput (Qi et al., 2019).

**Figure 1:**
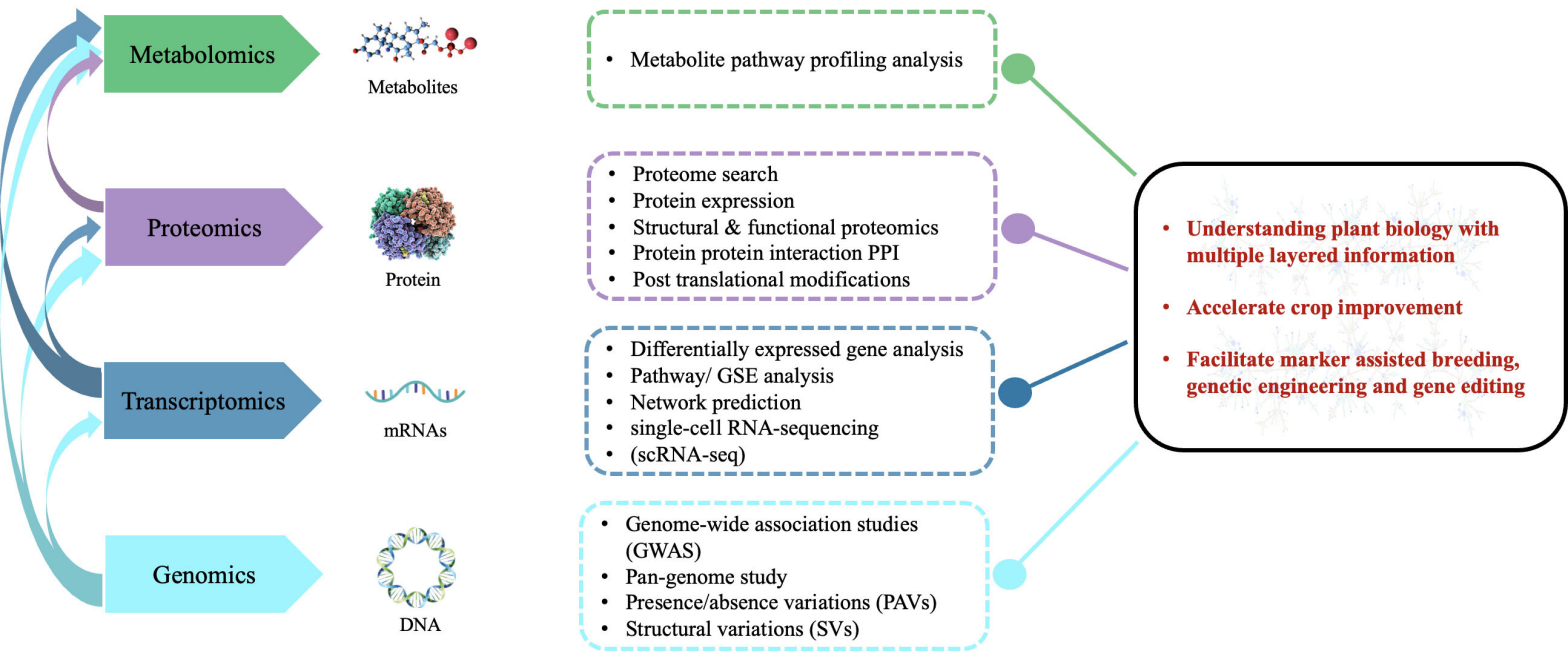
The prospects, limitations, and outlook for multi-omics based agricultural development in the future. Different multi-omics data layers, their interactions, the kinds of omics features found in each layer, and the methods used to evaluate omics data in various layers.

In addition, the integration of multi-omics data could interpret gene functions and networks better, under different biotic and abiotic stresses (**Figure 1**) (Yang et al., 2021). Using comprehensive multi-omics techniques, researchers have identified essential key factors in senescence, stress response, and harvest index of many economically important crops such as rice, wheat, soybeans, rapeseed, and maize (McLoughlin et al., 2018; Peng et al., 2020; Uchida et al., 2020; Ma et al., 2021a; Raza et al., 2021b). In the present review, we represent how the multi-omics revolution has upgraded plant breeding efficiency to enhance nutritional values, crop yield, and resistance against biotic and abiotic stresses of wild species for sustainable food security. In the future, the integration of multi-omics strategies will play an immense role in improving genetic development and crop breeding.

## 2 Multi-omics techniques accelerate the genetic dissection of complex traits

### 2.1 Genomics and pan-genomics

The relationship of genotype with phenotype is a prerequisite for accurate breeding design. Therefore, it is essential to genetically dissect the agronomic traits and identify the corresponding phenotypic variations. Numerous revolutions in DNA sequencing technology over the last 40 years have significantly improved sequencing throughput and quality continued to reduce costs, and significantly facilitated genome advancement and functional research (Shendure et al., 2017).

NGS is a deep sequencing (DP) or massively parallel sequencing (MPS) that permit plant genomes decoding. The first plant genome of *Arabidopsis* was constructed 22 years ago (Kaul et al., 2000; Theologis et al., 2000). Less than 300 whole genome assemblies at the chromosomal level (representing about 900 species) have been sequenced so far out of the estimated ~0.5 million species in the green plant clade (Kress et al., 2022), that include rice (Goff et al., 2002), maize (Schnable et al., 2009), tomato (The Tomato Genome Consortium, 2012), wheat (Ling et al., 2013) and rapeseed (Chalhoub et al., 2014). Benefitting from available high-quality reference genomes, a fairly large collection of genetic resources or populations of different crops are genotyped at the whole genome level. For example, it is estimated that whole-genome resequencing has so far been done for more than 6,000 soybeans accessions (Zhang et al., 2022b) and at least 10,000 rice accessions (Wing et al., 2018), which provide abundant genetic variation resources for genomic breeding of crops.

So far, advances in high-throughput sequencing technology have made the reference genome available in more than 800 plants (Shen et al., 2022), and most of which are *de novo* assembled by third-generation sequencing (TGS). The pan-genome was proposed first in 2005 and rapidly developed in recent twenty years due to the advent of Pacific Biosciences (PacBio) and Oxford Nanopore Sequencing (ONT) platforms. Compared to short insertions/deletions (indels) and SNPs, the structural difference (such as presence/absence variations (PAVs) and structural variations (SVs)) identified by pan-genome analysis play a vital role in the dissection of complex traits. The structural differences that have been determined in the pan-genome era requires a reassessment of the bases of phenotypes. The SVs have already been associated with environmental changes (Sutton et al., 2007; Cook et al., 2012) flowering time (Nitcher et al., 2013; Würschum et al., 2015), stress tolerance (Gabur et al., 2019), and plant domestication traits such as plant architecture (Tan et al., 2008; Zhou et al., 2009) and dehiscence (Lin et al., 2012).

It has been shown that SVs are associated with a wide range of biotic and abiotic stress tolerance in a various crop species. In maize, resistance to the sugarcane mosaic virus was induced at the *Scmv1* locus by a complex SV comprising several transposable elements (TEs) that changed the expression level of atypical h-type thioredoxin (Liu et al., 2017). In potato, resistance to late blight in was acquired by two genes *R1* and *ELR* that were introgressed from wild potato although missing in cultivated potato (Du et al., 2015). Furthermore, the graph-based pan-genome will revitalize the re-sequenced data in intercepting novel genetic variations, especially for large SVs (Shen et al., 2022). In PAVs of varied sizes and different lines of sorghum, the common deletion of a sulfotransferase gene conferred tolerance to the parasitic weed *Striga* by minimizing its germination stimulant effect (Gobena et al., 2017). Moreover, in abiotic stress resistance, variation in *HvCBF4* and *HvCBF2* copy number at the *FR-H2* locus was linked to frost tolerance in barley (Francia et al., 2016). Salt sensitivity in soybean was found to be caused by an insertion of a *Ty1/copia* retrotransposon in the *GmGHX1* cation H+ exchanger gene (Qi et al., 2014). The *Pup1* locus in rice has been associated with the presence of a receptor-like cytoplasmic kinase gene that confers tolerance to phosphorus deficiency (Gamuyao et al., 2012). These studies suggested that SVs, despite their narrow focus, are likely to significantly influence crop improvement in changing climate.

Recently, the pan-genomes of cucumber, soybean, and rice were analyzed using graph-based approaches, revealing numerous novel large SVs and considerably easing the identification of relevant genetic variations for certain complex traits (**Table 1**) (Liu et al., 2020; Qin et al., 2021; Li et al., 2022a). In-depth, the rice pangenome research has revealed that SVs and gene copy number variations (gCNVs) supported environmental response and artificial selection during the process of evolution and domestication (Qin et al., 2021). A prior study on 25 inbred lines of maize identified similarities between PAVs and heterotic group membership (Darracq et al., 2018), showing that SVs can be useful for characterizing heterotic groups and selecting parental lines for the development of hybrid crops. In general, the comprehensive genome composition heterogeneity outlined by genomes and pan-genomes facilitates the establishment of novel methodologies for plant scientists to investigate functional alleles for phenotypic variations and for breeders to increase genetic resources to enhance crop germ plasm in changing climates (Della Coletta et al., 2021; James et al., 2021).

**Table 1:** List of recently available Pan-genome/integrated databases with available tools.

Species	Accessions	Available tools/information	Database name	URL	Reference
**Banana**	15	RNA-Seq display, Synteny viewer, JBrowse, GO enrichment, Panache, MusaCyc, Gigwa, Locus converter, Primer Blaster & designer	BGH	https://banana-genome-hub.southgreen.fr/content/panache	Droc et al., 2013
**Citrus**	23	GWAS tool, JBrowse, CRISPR design, KEGG/GO enrichment, Pangenome Search, Gene ID Convert	CPBD	http://citrus.hzau.edu.cn/index.php	Liu et al., 2022c
**Maize**	26	JBrowse, GBrowse, NAM genomes, Newly Characterized, Pangenome Search, Genes, Metabolic Pathways, Phenotype/Mutant Data, qTeller	MaizeGDB	www.MaizeGDB.org	Hufford et al., 2021
**Rapeseed**	1689	GBrowse, KEGG/GO enrichment, Pangenome Search, Metabolic Pathways, Phylogenetic analysis, Blast sequence, Statistics of Homologous Regions	BnPIR	http://cbi.hzau.edu.cn/bnapus	Song et al., 2021
**Rice**	3010	GBrowse, Pangenome Search, Phylogenetic analysis, Blast sequence, PAVs, and expression profiles	RPAN	http://cgm.sjtu.edu.cn/3kricedb/	Sun et al., 2017
**Soybean**	204	GBrowse, Genetic Maps, RNA-Seq Atlas, Pangenome Search, Metabolic Pathways, Pedigrees Database, Genotype Comparison Tool (GCViT)	PanSoy	https://soybase.org/projects/SoyBase.C2021.01.php	Torkamaneh et al., 2021
**Tomato**	838	GBrowse, Browse QTLs, Pangenome Search, Alignment Analyzer, Motifs Finder, Biochemical Pathways, Ontology Browser, Expression database	SolOmics	https://solgenomics.net/projects/tgg	Zhou et al., 2022b
**Wheat**	16	Pangenome Search	Wheat Panache	http://www.appliedbioinformatics.com.au/wheat_panache	Bayer et al., 2022
**19 species**	46	Pangenome of 19 species available, Phylogenetic analysis, Gene family analysis	GreenPhylDB	https://www.greenphyl.org	Valentin et al., 2021

### 2.2 Epigenomics

During the last few decades, epigenomics has been developed into a frontier omics technique that can be used to explain the changes in the regulation of gene activities and expression under the epigenetic modifications of DNA sequences (Saeed et al., 2022). These epigenetic changes, such as DNA methylation, histone modifications, and chromatin accessibility, may assist us in determining how crops adapt to environmental changes without altering their DNA sequences.

Several techniques that can be used to investigate DNA methylation in plants, including whole genome bisulfite sequencing (WGBS), methyl-sensitive amplification polymorphism (MSAP), and methylated DNA immunoprecipitation (MEDIP) (Sun et al., 2022). The MSAP and WGBS techniques are frequently used to investigate the methylation status involved in regulating gene expression in plants under environmental stresses. Epigenetic research has revealed that drought-tolerant plants exhibited a more stable methylome, with differentially methylated regions (DMRs) linking genes primarily involved in the programmed cell death and stress response in mulberry (Ackah et al., 2022), mungbean (Zhao et al., 2022) and rice (Wang et al., 2016). Likewise, the differentially methylated epiloci (DME) identified 12 stress-related genes in rice genotypes under high temperature treatment (Li et al., 2022b). The methylome content of alfalfa plants increased during salinity stress, and 5-AzaC treatment (DNA methylation inhibitor) reduced the salt tolerance (Al-Lawati et al., 2016). Similarly, hypermethylation is one defense mechanism for plants that protects them from potential injury from heavy metal compounds and enables them to survive in harsh environments (Raza et al., 2022a; Sun et al., 2022). Numerous DNA-methylation studies have been conducted associating with seed development, plant organ differentiation, fruit ripening, and response to environmental stresses AND the functional role in gene regulation (Zhang et al., 2018). These studies provided evidence that DNA methylation may have regulatory roles in determining plant stress tolerance under a range of stress conditions.

In relation to plant growth and stress response mechanisms, histone changes and chromatin remodeling are important epigenetic processes that control gene expression by altering the chromatin status and activating transcription regulators. Most of techniques for detecting epigenome-level histone modifications rely on immunoprecipitation such as chromatin immunoprecipitation sequencing (ChIP-seq) and its modified form ChIP-exo, which has more specific binding sites. Other histone profiling techniques that are available and can be used effectively include ChIPmentation (ChIP-Tn5 transposase tagmentation), CUT&Tag (cleavage-under targets & tagmentation), and DamID (*E. coli* deoxyadenosinemethylase + protein of interest) (Mehrmohamadi et al., 2021). However, ChIP-seq is a commonly used technique in plants to identify the interaction between DNA-methylation sites and histone proteins (Chen et al., 2018). The dynamic variation in H3K4me3 and H3K27me3 in castor beans under salt stress was closely correlated with the transcript abundance of *RSM1*(*RADIALIS-LIKE SANT/MYB 1*), which has previously been identified as a positive regulator of salt resistance (Han et al., 2020). Following the post-harvest desiccation phase, the ABA-biosynthesis genes are activated in tea plants, resulting in ABA accumulation due to increased histone acetylation and decreased H3K9me2 (Gu et al., 2021). According to a recent study on rice, the dynamic nature of histone H3K27me3 and H3K27ac modifications regulates gene expression that are responsive to cold (Dasgupta et al., 2022). In addition to triggering broad changes in the histone methylome, stress signals specifically affect the methylation of genes responsive to stress (Xiao et al., 2022). These results demonstrate a correlation between stress-induced histone modifications and genome-wide transcription remodeling.

Chromatin accessibility profiling has been carried out in numerous plant species using DNase-seq (Dnase I hypersensitive sites sequencing), Mnase-seq (micrococcal-nuclease digestion sequencing), FAIRE-seq (formaldehyde-assisted isolation of regulatory element sequencing) and ATAC-seq (assay for transposase-accessible chromatin-sequencing) has found a wealth of novel information about the regulatory dynamics in plant genomes. Studies on chromatin accessibility have made it possible to build transcriptional networks that respond to environmental changes in rice (Wilkins et al., 2016). To illustrate the effect of abiotic stresses on *A. thaliana* two different techniques, FAIRE-seq and Dnase-seq were used to capture the open chromatin states (Raxwal et al., 2020). Moreover, chromatin accessibility analysis in maize cold tolerant lines during stress has revealed the re-allocation of resources from growth to defense (Jonczyk et al., 2020). To investigate the relationship between chromatin characteristics and gene expression in grapevine, chromatin accessibility was determined using ATAC-seq, Hi-C, and ChIP-seq (Schwope et al., 2021). Epigenetic changes are also involved in organ development and cell differentiation across the species (Maher et al., 2018). These studies contribute to our understanding of how plants respond to environmental cues by changing their gene expression, and how chromatin-based regulation of gene expression is probably essential for these responses.

### 2.3 Transcriptomics

Transcriptomics is the study of “transcriptome” which refers to the entire collection of RNA transcripts generated by an organism’s genome in a cell or tissue (Zhou et al., 2022a). Transcriptomics encompasses both mRNAs and ncRNAs in the cells, and it has recently been used extensively in crop breeding to investigate gene expression under different conditions (Schiessl et al., 2020). Traditional profiling approaches, which include differential display-PCR (DD-PCR), cDNAs-AFLP, and SSH were initially used to assess transcriptome dynamics, but these methods had poor resolution (Nataraja et al., 2017). The emergence of advanced throughput sequencing has allowed plant scientists to conduct extensive transcriptomics research (Mir et al., 2019). Recently, RNA-seq employing NGS methods has made it possible to characterize the transcriptome more precisely than with microarray.

The differential expression of mRNAs in rapeseed during development stages was determined to find the candidate genes controlling seed size (Niu et al., 2020). Signal peptides have been linked to the regulation of many plants’ biological processes, including immune response and development, according to the rice transcriptome study (Wang et al., 2020a). A comparative transcriptomic analysis of two extreme Chinese cabbage genotypes revealed that ion homeostasis is a significant biological pathway associated with plant’s instant adaptation to salt tolerance (Li et al., 2021a). It was also discovered that the transcription factors MYB, bZIP, and WRKY serve as regulators in the salt-responsive signaling pathway of maize roots (Zhang et al., 2021a). Similarly, genome-wide transcriptome profiling showed that the apple’s WRKY gene family responded to various biotic stresses (Zhang et al., 2021b). Transcriptome studies also include various kinds of non-coding RNAs. These non-coding RNAs come in many forms, including micro RNAs (miRNAs), circular RNAs (circRNAs), long non-coding RNAs (lncRNAs), ribosomal RNAs (rRNAs), and short interfering RNAs (siRNA), and they thought to be a promising target for crop improvement (Waititu et al., 2020; Zhou et al., 2022a). These non-coding RNAs perform various functions, such as miRNAs involved in slicing and post-transcriptional modification of target mRNAs. While lncRNAs serve as important regulators in several vital biological processes and circRNAs functioned as miRNA sponges, transcriptional and splicing regulators, and moderators of primary gene expression in plants (Lai et al., 2018; Waititu et al., 2020). Whole-transcriptome sequencing in plants was used to create the global landscape of these mRNAs in plants, such as maize (Liu et al., 2022a) Chinese cabbage (Shi et al., 2022) citrus (Fu et al., 2019).

Since it is generally recognized that different cell types play different biological functions in plant growth and development, it has become critically important in recent years to examine the transcriptome responses of plants at cellular level. In molecular biology, single-cell RNA-sequencing (scRNA-seq) is a high-resolution method is growing in popularity for studying plant functional genomes and transcriptional activity at the single-cell level. This method enabled researchers to examine heterogeneity in plants within different cell types (Yaschenko et al., 2022). The polyploidization events in plants were poorly understood by traditional transcriptome studies. However, the advent of scRNA-seq enabled to study of female gametes cells (Arabidopsis) without cross-contamination, advancing plant hybridization, polyploid genetics, and reproductive biology (Song et al., 2020). While scRNA-seq technologies have been extensively used in animal science, their application in plant sciences has just recently been understood. Hence, there have been fewer studies on the high-throughput single-cell transcriptome in plants than in animals until now. Thereafter, this knowledge could be put to better use in breeding initiatives with innovative and targeted goals to assist, for instance, crop production and quality, plant climate change adaptations, and plant tolerance to biotic and abiotic stressors.

### 2.4 Proteomics

Proteomics is a technique for analyzing all the proteins expressed within an organism, and it splits into four major types: sequence, structure, function, and expression proteomics (Aizat and Hassan, 2018). Different approaches are used to analyze these types, for intence, sequence proteomics analyzed by HPLC (high-performance liquid chromatography) (Hao et al., 2018) and structural proteomics by nuclear magnetic resonance (NMR), electron microscopy, crystallization, and X-ray diffraction of protein crystals (Woolfson, 2018; Opdensteinen et al., 2022). However, functional proteomics is examined through various methods as yeast one (Y1H) or two hybrids (Y2H) and protein micro-array profiling (Yang et al., 2020; Mao et al., 2020).

The development of protein extraction and separation techniques at the sample and genome-wide level has led to a rapid breakthrough in plant proteome research. Traditional proteomics methods rely on chromatography-based approaches. However, enzyme-linked immunosorbent assays (ELISA) could be used to study specific proteins. Subsequently, some advanced gel-based techniques were developed for protein quantification such as SDS-PAGE, 2-DE (two-dimensional gel-electrophoresis), and 2D-DIGE (two-dimensional differential gel-electrophoresis) (Yang et al., 2020).

In addition, protein microarrays and chips have been developed to analyze protein expression in small amounts of protein samples efficiently. Likewise, more hi-tech methods for quantitative proteomic analysis have been developed, including isotope-coded affinity tag (ICAT) labeling, stable isotope labeling with amino acids in cell culture (SILAC), and isobaric tag for relative and absolute quantification (iTRAQ) (Yang et al., 2020). The three-dimensional structure of proteins can be determined using a high-throughput technique called NMR spectroscopy, which may help to explain how proteins work biologically (Xiang et al., 2018). Despite the recent development of single-cell sequencing technologies to study the mechanism of cellular activity, single-cell proteome quantification still lags single-cell transcriptome achievements (Kashima et al., 2020; Vistain and Tay, 2021). However, Mass spectrometry (MS) has been evolving recently to measure the single-cell protein level (Vistain and Tay, 2021).

All the proteomic technological achievements could help scientists study the environmental effects of protein modifications in terms of crop resilience (Zhang et al., 2022a). Numerous studies have used proteomic analysis of the genetic complements of proteins to measure proteins and identify changes in protein expression as they relate to crop response to abiotic stimuli (Ueda and Seki, 2020; Zhang et al., 2022a). So, it might be possible to identify protein-regulatory mechanisms and understand how a particular protein contributes to stress tolerance by examining the proteome differences in crop responses to abiotic stimuli (Yu et al., 2018). This can be applied to crop breeding to increase agricultural outputs in the future even in challenging environmental conditions (Schulze et al., 2021).

### 2.5 Metabolomics

Metabolomics is one of the most current omics technologies for probing metabolites and elucidating crop resilience. According to the plant study trials, the inclusion of untargeted metabolome detection sped up the development of integrated metabolomics (Kumar et al., 2021). Primary metabolites are required for the synthesis of lipids, sugars, and amino acids in plants however, secondary metabolites comprised of flavonoids, atropine, carotenoids, phytic acid, as well as ROS, coenzymes, and antioxidants (Razzaq et al., 2019). Since metabolites are typically the main product in complex metabolic cascades, they can link the phenotype with the genome, transcriptome, and proteome (Misra et al., 2018).

The plant kingdom contains at least 391000 unique metabolomes (Nakabayashi and Saito, 2020); therefore, the development of analytical techniques is required to determine as many specialized metabolomes as possible. Analyses that are either targeted or untargeted can be used to assess the relative and absolute levels of metabolites. The analytical methods used for metabolomics primarily comprised liquid chromatography-mass spectrometry (LC-MS), gas chromatography-mass spectrometry (GS-MS), capillary electrophoresis-mass spectrometry (CE-MS), HPLC (high-performance liquid chromatography), NMR, and direct flow injection (DFI) (Razzaq et al., 2019; Raza, 2022).

NGS technologies have emerged as effective tools for examining gene regulation and the molecular dynamics of plant cellular responses to biotic and abiotic stresses (Abdelrahman et al., 2018). However, it is now possible to infer an early metabolic network from an organism’s genomic sequence by integrating metabolomics and NGS (Pan et al., 2020). The integrated information obtained from the NGS and metabolites profiling could help us to improve the crops in changing climatic conditions (Scossa et al., 2021). In the presence of numerous biotic stresses, the crucial function of metabolites in cereal crops, such as maize, barley, and rice has been recognized (Yang et al., 2021). The integrated transcriptome and metabolomic analysis of sesame emphasized the significance of amino acid metabolism, especially the saccharopine pathway, ABA metabolism, and signaling pathway for drought resistance (You et al., 2019). The identification of several candidate genes and metabolites associated with isoflavone synthesis and the tricarboxylic acid cycle further supports the significance of these metabolic pathways in the response of soybean to drought (Wang et al., 2022). Similarly, the role of metabolites (phenolics and phenylpropanoids) has been identified in maize under biotic and abiotic stresses (Block et al., 2020). In tomato, *Trichoderma*-induced biotic stress resulted in various metabolome alterations, which revealed that treated plants accumulated more isoprenoids (Coppola et al., 2019). In addition to stress resistance, metabolomics research in combination with other omics platforms is important for crop nutritional values that can provide adequate insights about the quality-related genes to develop future crops (Pott et al., 2021). These examples demonstrate how omics research can define complex molecular relationships. Therefore, metabolome studies provide an integrated depiction of numerous activities extending from the genome to metabolome as well as phenotypic traits when combined with other omics data including genomics, transcriptomics, and proteomics.

## 3 Multi-omics data integration for crop improvement

Crop improvement, including high yield and tolerance to biotic and abiotic stresses, is a long-term and time-consuming process in current plant breeding. The development of omics techniques and the rapid accumulation of omics data provide possible solution and foundational information. Approaches to integrating multi-omics information are being leveraged in plants for a better understanding of molecular mechanisms underlying complex traits and acceleration of the crop improvement (**Figure 3**). Here, we discuss in detail how to apply multi-omics strategies or tools to dissect the genetic basis/architecture of complex traits and predict key agronomic phenotypes in crops.

### 3.1 Genetic dissection of complex traits using multi-omics data

To dissect the genetic architecture of complex traits, candidate gene mining is an essential step. In the early days, gene mapping is usually difficult due to the lack of a high-quality crop genome. Luckily, the number of crop genome and pan-genome have increased dramatically in the past 20 years, bridging the genotype–phenotype gap easy. The most widely used strategies to identify genes responsible for complex traits are linkage mapping studies in family-based populations, association mapping in natural plant population, joint linkage-association mapping using both bi-parental and natural populations, and genomic selection between diversification population produced by plant domestication. In linkage analysis, genetic maps are usually constructed based on segregating mapping populations, such as doubled haploid (DH) lines, F2 populations, recombinant inbred lines (RILs), and backcross inbred lines, is to be used for quantitative trait locus (QTL) mapping. As an alternative to conventional linkage analysis, bulk segregation analysis (BSA) (Gao et al., 2022) coupled with next-generation sequencing (NGS) can be used in the genetic mapping of simple qualitative traits controlled by major genes when two sample pooling with extreme phenotypes exists.

For association analysis, we usually exploit genetic variation of the whole-genomic level in natural populations, to use linkage disequilibrium (LD) to map genes that cause a specific phenotype. In addition, a gene-based association approach, transcriptome-wide association studies (TWAS) can be used to investigate gene-trait associations using genetically regulated gene expression (Wainberg et al., 2019). The genes selected during domestication and artificial selection play an important role in modern crop breeding. Genome-wide selective sweep analysis is usually used to identify the major genes that enhance environmental adaptation (Lu et al., 2019; Li et al., 2021b), involved in morphotype changes (Kang et al., 2021), and selection for key agronomic traits (Hu et al., 2022).

**Figure 2:**
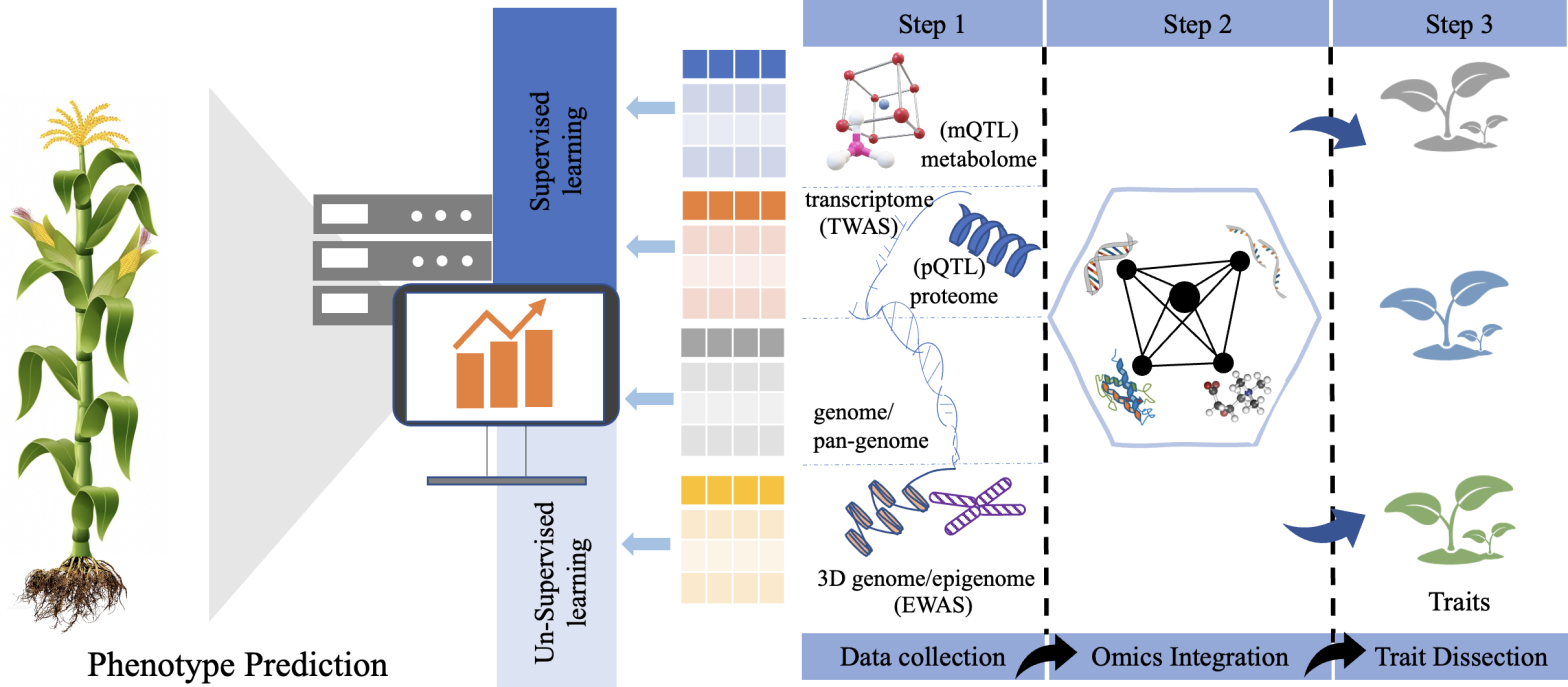
Strategies of multi-omics data integration between different layers of omics data to dissect the complex agronomic traits and phenotype prediction by using machine learning methods.

However, gene identified as part of functional genomic studies, is just the beginning of the genetic dissection of complex traits. Its major disadvantages are: (1) The number of genes identified by QTL mapping is far beyond experimental expected. (2) It’s difficult to directly infer the causal relation between genes and final phenotypic traits due to the lack of evidence of middle omics. To address it, the integrating genomics with other omics data sets will reduce further candidate genes and benefit system analysis of gene function. Molecular phenotype is defined as a collection of molecular characteristics (i.e., mRNA transcripts, proteins, and metabolic compounds) in central dogma (Pramanik et al., 2020), which can be used for deep phenotyping. These phenotypic data permit a better understanding of end-to-end mechanistic from genes to final traits in crops (**Figure 2**). Recent advances in omics techniques allow for producing many molecular phenotypes at a larger-scale population level, making the association implementation of multi-omics molecular phenotypes easy (**Table 2**).

**Table 2:** Recent GWAS studies combined with RNA, protein, and metabolites to find the target traits in breeding programs.

Multi-omics approach	Crops	No. of accessions used	Target trait	References
**EWAS**	Maize	263	Development stages	Xu et al., 2019
**EWAS**	Maize	51	Adoptive evolution	Xu et al., 2020
**EWAS**	Palm	31	Reproductive defect	Ong-Abdullah et al., 2015
**EWAS**	Soybean	45	Domestication history	Shen et al., 2018
**EWAS**	Wheat	104	Adaptation/stress	Gardiner et al., 2018
**TWAS**	Cotton	251	Fiber	Li et al., 2020
**TWAS**	Cotton	216	High temperature	Ma et al., 2021
**TWAS**	Maize	323	Leaf architecture	Lin et al., 2022
**TWAS**	Maize	~1500	Tocochromanols	Wu et al., 2022
**TWAS**	Maize	299	Kernel traits	Kremling et al., 2019
**TWAS**	Millet	398	Metabolic traits	Li et al., 2022c
**TWAS**	Rapeseed	505	Seed oil content	Tang et al., 2021
**TWAS**	Rice	305	Glycemic index and texture	Anacleto et al., 2019
**TWAS**	Sorghum	869	Intrinsic water use efficiency	Ferguson et al., 2021
**TWAS**	Tomato	610	Fruit ripening	Zhu et al., 2018
**TWAS**	Tomato	580	Fruit and pathogen	Szymañski et al., 2020
**PWAS**	Maize	Natural population (ZHENG58 × SK RIL)	Kernel traits	Zhou et al., 2021a
**MWAS**	Millet	360	Grain quality	Li et al., 2022c
**MWAS**	Mellon	44	Taste and flavor	Moing et al., 2020
**MWAS**	Rice	533	Agronomic traits	Wei et al., 2017
**MWAS**	Soybean	398	Seed oil content	Han et al., 2022
**MWAS**	Tomato	610	Fruit quality/ripening	Zhu et al., 2018
**MWAS**	Tomato	76	Leaf vs other tissues	Nunes-Nesi et al., 2019
**MWAS**	Tomato	76	Seed metabolites	Alseekh et al., 2020
**MWAS**	Tomato	580	Fruit and pathogen	Szymañski et al., 2020
**MWAS**	Tomato	107	Steroidal alkaloid	Dzakovich et al., 2022
**MWAS**	Wheat	182	Kernel traits	Chen et al., 2020

EWAS, Epigenome-wide association studies; TWAS, Transcriptome-wide association studies; PWAS, Proteome-wide association studies, and MWAS, Metabolome-wide association studies

### 3.2 GWAS + epigenome-wide association studies (epi-GWAS or EWAS)

Epigenome-wide association studies (EWAS) is a powerful method for identifying traits-associated epigenetic variation, specifically variation in DNA methylation, which has been used in human studies (Rakyan et al., 2011; Hawe et al., 2022). The integration of EWAS (i.e., methylation quantitative trait loci (meQTL)) with GWAS can help us to illuminate functional mechanisms underlying genetic variant-trait associations (Huan et al., 2019). However, there are still few EWAS applications based on the large-scale population level although the studies on GWAS have been widely used in plants, which present novel opportunities but also create new challenges for future crop improvement.

### 3.3 GWAS + transcriptome-wide association studies

GWAS and transcriptome-wide association studies (TWAS) are renowned techniques for locating genomic regions or genes for which DNA sequence or gene expression variations are associated with quantitative variability in a trait of interest (Ferguson et al., 2021). TWAS is a useful tool to complement GWAS since it explores the relationships between variations in transcript abundance and phenotypic variance. The most recent use of Fisher’s combination test to integrate GWAS and TWAS offered proof-of-concept by showing how it improved the efficiency with which identified associated genes could be “re-discovered” for well-known maize kernel traits (Kremling et al., 2019).

GWAS-implicated genes were further refined using expression QTL (eQTL) analysis through TWAS as a fine-mapping technique to find candidate genes. To identify the key genes affecting both the glycemic index and cooking properties of rice, a similar approach was used to determine the amylose content and ultimate stickiness (Anacleto et al., 2019). Cotton eQTL mapping was employed in a prior study to connect the regulatory variations to gene expression in fiber formation (Li et al., 2020). TWAS creates a direct relationship between gene expression and phenotype utilizing eQTL data, as contrasted to the functional study of leading SNPs or homology-based identification of relevant genes for GWAS locations (Zhu et al., 2018). These studies provided evidence that examining the intermediary transcriptome between variome and phenome can help us better understand the regulatory functions of genetic variations driving phenotypic change.

### 3.4 GWAS + proteome-wide association studies

Protein QTL (pQTL) analysis, which has produced proteome networks for clinical applications, has been used extensively in epidemiological research (Suhre et al., 2017), but it is still rarely used in plant-based GWAS studies. Moreover, it is important to comprehend the functional contexts of gene expression variation through modern crop breeding (Jiang et al., 2019). Recently, pQTL mapping of maize inbred lines was studied using an integrated multi-omics approach to identify the candidate genes associated with kernel size (Zhou et al., 2021a). These findings revealed that genes involved in signal transduction, amino acid metabolism, and an unidentified mechanism may control maize kernel size. Protein abundance variation in crops gains a new level of functional context owing to an integrated multi-omics approach combining pQTL analysis. The use of protein-based GWAS studies in crop breeding is still limited and needs to be utilized to discover the hidden candidates to improve the breeding programs.

### 3.5 GWAS + metabolome-wide association studies

Metabolite-based genome-wide association studies (mGWASs) or metabolite QTL (mQTL) made it possible to concurrently screen a large number of accessions for possible associations between their genomes and various metabolites and can provide insights on the genetic basis of complex traits and metabolic diversity (Peng et al., 2017; Fang and Luo, 2019; Li et al., 2022c). To discover new associations between genes and metabolites, mGWASs has been effectively used on several model plants and agronomic crops (Zhu et al., 2018; Zeng et al., 2020; Li et al., 2022c). The mGWAS analysis discovered notable variations in the core genetic architecture and the natural variability of the metabolites between different subgroups of foxtail millet (Li et al., 2022c). The degree of natural variability in metabolism and its genetic and biochemical regulation in tomatoes have been extensively elucidated by the integration of metabolomics, linkage mapping investigations, and mGWAS (Zhu et al., 2018). The metabolic breeding history of the tomato was recently discovered by an integrated analysis of eQTL and mQTL (Zhu et al., 2018). Understanding the mechanisms driving the evolution of metabolism is made possible by the mGWAS investigations. Despite the great progress that has been made in the mGWAS research, comprehensive understandings of metabolic control are still rare.

In addition, integration of eQTL, pQTL, and/or mQTL can be used to predict the quality trait to facilitate breeding as in the phenotypic prediction, multi-omics data modeling can explain that how a final phenotype is controlled by the differential expression of mRNA, protein, and metabolite in the central dogma. Similarly, Xu et al. (2016) found that the predictability of hybrid rice yield can be further increased by using these omics data. An earlier investigation utilizing potato tubers has demonstrated how a particular trait is related to gene expression, protein profiles, and metabolite data using the random forest regression method (Acharjee et al., 2016). These omics platforms can help us to develop strategies for integrating these omics data sets to forecast phenotypic features. This results in networks with relatively small sets of interlinked omics variables that are better able to predict the desired trait.

### 3.6 Exploiting multi-omics data for phenotypes prediction

Integrating data from multiple sources to create a model that can be used to predict complex traits and improve predictive accuracy is imperative. To date, an increasing number of statistical models including linear and nonlinear models, have been developed and widely used in phenotypes prediction. Genomic Best Linear Unbiased Prediction (GBLUP) is a linear model, which has been used extensively for genotype to phenotype prediction. To identify optimal prediction models, Wang et al. (2019) and Xu et al. (2016) have demonstrated that GBLUP generally yielded better results than other prediction methods using multi-omics data for selecting hybrid rice. Recently, Hu et al. (2021a) built a two-kernel linear model for multi-omics prediction of oat agronomic and seed nutritional traits in multi-environment trials and distantly related populations, show greater prediction accuracy than GBLUP. Similarly, as an extension of linear models, Linear mixed models (LMMs) have great potential multi-omics data prediction analysis (Weissbrod et al., 2016). However, a large amount of noise presented in high-dimensional omics data will limit predictive power of LMM. To filter out the noise, several novel methods were produced by extending the standard LMM and combining them with other prediction models (e.g., Bayesian sparse linear mixed model (BSLMM) (Zhou et al., 2013) and penalized linear mixed model with generalized method of moments estimator (MpLMMGMM) model (Wang and Wen, 2022)) have been proposed for modeling multi-omics data.

In nonlinear methods, machine learning (ML) is a new programming paradigm that can learn statistical rules from large-scale complex data, providing a scalable and interpretable framework for multi-omics integration and is usually applied to phenotypes prediction of crops in practice. The basic ML model can be divided into two major types supervised learning and unsupervised learning (Wang et al., 2020b). In supervised learning, classification (e.g., quality traits) and regression (e.g., quantitative traits) are the two major tasks to be predicted. Through the integration of multi-omics data using random forest, Acharjee et al. (2016) perform prediction of four quality traits in potato, including tuber flesh color, differential scanning calorimetry (DSC) onset, tuber shape and enzymatic discoloration. Deep learning (DL) is a specific subfield of machine learning which extensively used in the life science and health field for high-dimensional multi-source data integration and phenotype prediction (Chaudhary et al., 2018). Although DL has been rarely reported in multi-omics integration studies of the crop, it still has great potential and advantages for crop breeding in the future. Unsupervised learning is mostly used to seek the representations in data, such as clustering, association, and dimensionality reduction (DR). Among these, DR plays an important role in high dimensional biology due to reducing the number of random variables to consider. For instance, the algorithms of DR applied in maize contribute to the development of multi-omics data association studies (Liu et al., 2022b).

ML has revolutionized plant research to analyze and interpret large phenotypic data sets, as it is now possible to measure and correlate genotypic to phenotypic data at different levels. In addition, ML also made it possible to access large amounts of high-throughput data and solve problems in pertinent domains by using freely available software, and algorithms such as DL in prediction of protein structures (Senior et al., 2020). However, there are still many challenges such as large data sets, different species, genotypes, phenotypes, and variable environments, and the heterogeneous and fragmented nature of the data (van Dijk et al., 2021). Contrary to ML modules, statistical approaches were used to predict genotype to phenotype in traditional approaches. These approaches have been very effective since they produce good estimation values (p-value), easy to interpret and not complicated to use as compared with ML modules. However, similar issues are also addressed in advanced ML research, and solutions are being discovered in the plant research field (Azodi et al., 2020) to expand the multi-omics research in variable environments.

## 4 Challenges and future perspective

### 4.1 Environmental challenges

The pressure on agricultural systems worldwide is rising owing to the world’s exponentially increasing population, which is expected to hit 10 billion in the next 30 years (Scossa et al., 2021). The rising global temperature, which is expected to increase by 1.1 to 5.4°C by the end of this century, is also a challenge for agriculture (Tollefson, 2020). These frightening projections indicate that crops would experience heat stress and a rise in the frequency of droughts during their growing seasons (Rustgi et al., 2021; Raza et al., 2022b). Furthermore, climatic changes will probably increase the intensity of both single and combination abiotic stresses, including drought (Shahzad et al., 2021), cold, heat, salinity, and submergence (Anwar et al., 2021). Abiotic stresses are expected to worsen with the predicted climate-change scenarios by increasing the prevalence and severity of insects, pests, and pathogens as well as weed species proliferation and beneficial soil bacteria, as well as endangering essential plant pollinators (Zenda et al., 2021). The world’s most resource-constrained and populous regions would endure malnutrition and food insecurity due to the lower food yield brought on by these changes (Molotoks et al., 2021). Even though resistant and high-yielding varieties have evolved into intensive farming methods, it is crucial to consider the probability that crop yields, land use, and food demand will increase in the future (Voss-Fels et al., 2019).

**Figure 3:**
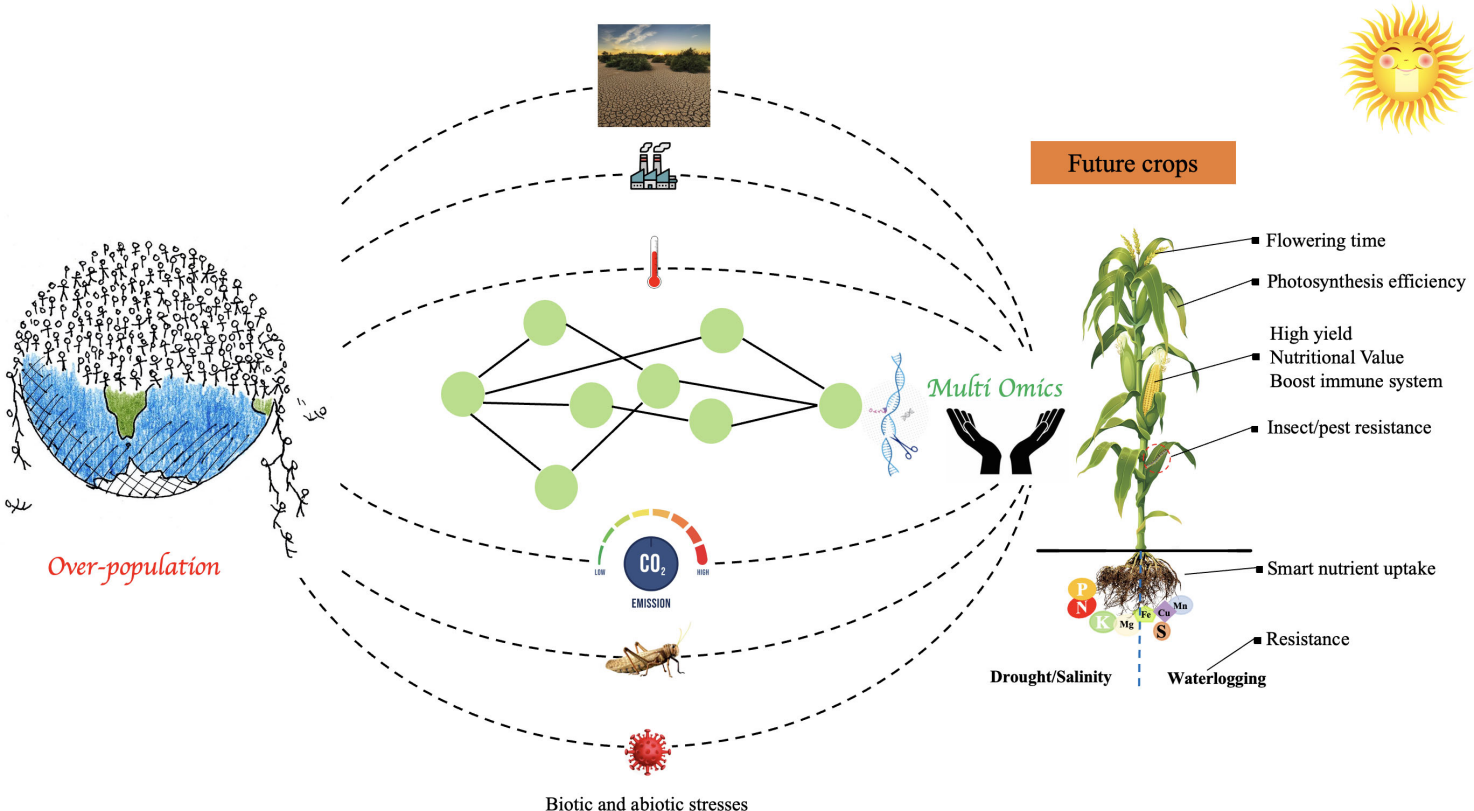
Schematic representation of the importance of integrated omics techniques in accelerating the development of future crops to feed the exponentially growing population. The development of climate-resilient and nutrient-rich crops is facilitated by integrated multi-omics approaches paired with genetics approaches, improved plant breeding, gene editing, and computational modeling techniques in a systems biology approach.

Plant breeders are putting forth innovative strategies to deal with the growing need for food grains in the context of environmental problems such as rising global temperatures, irregular rainfall patterns, and concurrent changes in pest and disease attacks (**Figure 3**). Resource depletion (land degradation, nutrient insufficiency, water availability) makes it even more important to enhance agriculture sustainably and reduce its CO_2_ emission (Mir et al., 2022). Increased temperatures and higher CO_2_ levels, which are connected to the impoverished nutrient density of several staple crops, exacerbate the serious health issues encountered by billions of malnourished people in low-income regions (Macdiarmid and Whybrow, 2019). Plant breeders have developed better cultivars of various crop plants to achieve these goals, generally by utilizing traditional plant breeding techniques involving genetic crossing and selection for the desired features. However, this approach chiefly focused on the crop’s major gene pool (Kaiser et al., 2020). Recent developments have made it possible for molecular plant breeding to now uses integrated multi-omics approaches, allowing plant breeders to insert desired genetic variations in the crop genome from a larger gene pool with better accuracy and speed. Hence, molecular plant breeding techniques are being aggressively added to the traditional crop improvement methods to efficiently get the desired results (Mir et al., 2022).

### 4.2 Challenges in multi-omics data integration

#### 4.2.1 Data archiving and sharing

There is an increasing need for integrated, rigorous omics research, like many other scientific domains. Data archiving is crucial for the repeatability of both individual and integrated omics datasets, as well as for adhering to Findability, Accessibility, Interoperability, and Reusability (FAIR) principles (Krassowski et al., 2020). The demand for open sharing of scripts and codes for these analyses (MATLAB, Java, R, and Python, etc.) *via* websites like GitHub (https://www.github.com), where developers can share code, review code, manage projects, and develop software alongside other developers, is a potential solution. High-throughput molecular abundance data, primarily gene expression data, are archived and publicly distributed *via* the Gene Expression Omnibus (GEO) collection at the National Center for Biotechnology Information (NCBI) (Rangwala et al., 2021). There are a few integrated omics databases (**Table 1**) available that can help researchers to better comprehend the flow of genetic information (RNA, protein, metabolite and phenotype) of which a trait is influenced.

#### 4.2.2 Data interpretation

The biggest challenge for omics datasets is the understanding of that large datasets. Biomarker discovery is one of the main goals of multi-dimensional omics approaches; regardless of the omics layer from which the key molecules are derived, the specificity and sensitivity of molecular biomarkers are crucial for the application of findings to breeding research and their practical application. Complex multi-layered network interpretation and curation are arduous, computationally, and time-consuming and demand in-depth knowledge of the biological system under study. Studies using an integrated omics approach frequently choose significant biological pathways and processes for hypothesis testing that are not physiologically relevant without applying a biological understanding of the system. Being familiar with understudied biological systems is crucial since it takes time and is often challenging to integrate the verification of datasets and networks (genes, proteins, and metabolites) for key biological pathways or mechanisms.

### 4.3 Future crops thanks to integrated multi-omics

#### 4.3.1 Crops with stress resistance

Several potential solutions to these issues have been put forth, including the development of climate-resilient crops, increasing the efficiency with which natural resources are used, diversifying agricultural systems, and linking agricultural intensification to the preservation of natural ecosystems (Peng et al., 2020). The most efficient approach to adapt our agricultural system to handle climate change is to design crops with desired agronomic traits that are climate resistant (**Table 3**) (Kumari et al., 2020) to that specific environment and demand. Omics approaches now enable developing elite climate-smart cultivars with desired traits like high yield, abiotic and biotic stress tolerance, and nutritive quality (Naeem et al., 2022; Reimer et al., 2022).

**Table 3:** List of stress (abiotic and biotic), nutrition, quality, and post-harvest related QTLs for future crop improvements.

Stress/Trait	Crops/Plants	QTLs	Reference
**Cadmium toxicity**	Rice	*qCd-7* and *qCdT7*	Liu et al., 2019
**Chilling**	Rice	*qLTSS3-4* and *qLTSS4-1*	Schläppi et al., 2017
**Cold tolerance**	Rapeseed	*qLTGA9-1* and *qLTGC1-1*	Zhu et al., 2021
**Drought**	Maize	*qWS-GY3-1* and *qWW-GY3-1*	Hu et al., 2021b
**Drought**	Rice	*qDTY2.4, qDTY3.3, qDTY6.3, qDTY11.2, qDTY1.1* and *qDTY8.1*	Yadav et al., 2019, Vikram et al., 2012
**Drought**	Rice	*qDTY 1.2, qDTY 2.2* and *qDTY 1.3*	Sandhu et al., 2014
**Drought/Grain Yield**	Wheat	*qGYWD.3B.2*	Shukla et al., 2015
**Salinity**	Maize	*qSFS1, qFFS1, qFDS1, qRLR1*, and *qFLR1*	Luo et al., 2019
**Salinity**	Rice	*qSL7*	Jahan et al., 2020
**Salinity**	Rice	*qSES1.3, qSL1.2, qRL1*, and *qFWsht1.2*	Rahman et al., 2017
**Salinity**	Wheat	*QPh-2D, QPh-4B* and *QPh-6A*	Luo et al., 2021
**Waterlogging**	Barley	*QTL-WL-4H*	Zhang et al., 2017a
**Zinc and Iron toxicity**	Rice	*qSdw3a, qSdw3b, qSdw12* and *qSFe5/qSZn5*	Zhang et al., 2017b
**Bacterial Wilt**	Chili	*Bwr6w-7.2, Bwr6w-8.1, Bwr6w-5.1, Bwr6w-6.1*, and *Bwr6w-7.1*	Lee et al., 2022
**Mildew**	Cucumber	*pm2.1, pm5.1, and pm6.1, dm2.1, dm5.2*, and *dm6.1*	Wang et al., 2018
**Sclerotinia stem rot**	Rapeseed	*qSRA2, qSRA3b* and *qSRC8*	Wu et al., 2019
**Nutritional value**	Rice	*qPr1, qPC1, qZn.1, qMn.1, qCa1-1, qFe1.1, qCo.1*, and *qAA.1* and *qSr.2*	Mahender et al., 2016
**Oil/Protein**	Soybean	*qOil-5-1, qOil-11-1*, and *qPro-14-1*	Huang et al., 2020
**Oil content**	Rapeseed	*Oil-A2-1-EJ* and *Oil-A5-1-DE*	Rout et al., 2018
**Postharvest**	Apple	*QTL Z16.1*	Wu et al., 2021
**Postharvest**	Lettuce	*qSL4*	Sthapit Kandel et al., 2020
**Postharvest**	Soybean	*qPS-DS16-1* and *qPS-DS16-2*	Seo et al., 2020
**Phosphorus efficiency**	Maize	*q14-2, q15-2*, and *q19-2*	Li et al., 2016
**Phosphorus efficiency**	Wheat	*QSpute-4B.2 and QTpute-4B.2*, and *QTpute-4B.1*	Yuan et al., 2017
**Pod shattering**	Rapeseed	*qSRI.A06* and *qSRI.A09*	Liu et al., 2016
**Starch**	Rice	*qRS7-1* and *qRS7-2*	Selvaraj et al., 2021
**Vitamin E, C and Carotene**	Tomato	*Vitc10.1, vitc8.1, vite5.2*, and *βcrn11.1*	Gürbüz Çolak et al., 2020
**Yield**	Rapeseed	*cqSGC–C2, cqSOC–A5–3*, and *cqSPS–A7–2*	Zhou et al., 2021b

To accelerate genetic advancement and reduce the effects of climate change on crop yield, advanced integrative breeding platforms are required (Bevan et al., 2017). A single reference genome cannot provide the full range of genetic variation needed for crop breeding. Therefore pan-genome research could help us to understand the genome composition of the population, whether cultivated, landrace, or wild progenitors. The PAVs in various species have been identified by a variety of biotic stress-responsive genes (Cook et al., 2012). The pan-genome of *B. napus* was examined to characterize disease-resistant genes, and 106 potential QTLs associated with blackleg resistance were found recently (Dolatabadian et al., 2020).

The developmental processes, epigenetic markers like histone modifications and DNA methylation influence how plants respond to environmental signals. The patterns of DNA methylation and subsequent differential expression of genes related to stress have all been shown to be influenced by a variety of biotic (Correa et al., 2020) and abiotic stress conditions such as water stress, nutrient deficiency, temperature stress, as well as *in vitro* stresses (such as tissue culture) (Scossa et al., 2021). If epigenetic processes also control plant phenotypic variation, then at least in part, hence epialleles should be considered when developing crop enhancement strategies.

On the other hand, meiosis has the potential to transfer stress-induced DNA methylation states, leading to some types of transgenerational memory (Dar et al., 2022), even though paramutation-like activities might complicate inheritance patterns (Minow et al., 2022). The availability of several important natural or induced epigenetic variations for agriculture, such as rice, maize, and soybean (Lloyd and Lister, 2022), demonstrates how screening epialleles can significantly increase the genetic variation that breeders can exploit to improve agricultural practices.

Moreover, integrated transcriptome and metabolome studies revealed that canola seedlings could survive the impacts of alkaline-salt stress by controlling the metabolism of organic acids and amino acids in roots (Wang et al., 2021). Another study has demonstrated that *ZmUGT* genes may control SA (salicylic acid) homeostasis at transcriptional and metabolic levels, contributing to maize pathogen defense response (Ge et al., 2021). Integrated transcriptome and proteomic datasets to identify pathways driving *S. subterranea* resistance in potato roots were examined, and this multi-omics approach discovered an increase in glutathione metabolism at the RNA and protein levels in the resistant cultivars (Balotf et al., 2022). Therefore, integrated omics techniques enable detailed comprehension of complicated physiological and molecular mechanisms underlying several crucial traits of agronomic value (Singh et al., 2020), integrating large molecular datasets and the developing predictive models for those key traits (Scossa et al., 2021). Developing climate-smart cultivars require a comprehensive, critical, and effective approach (Pazhamala et al., 2021). When it comes to generating climate-ready crops, these multi-omics-generated data will need to be combined with contemporary plant breeding and gene editing techniques (Gogolev et al., 2021). These high throughput sequencing technologies and integrated multi-omics platforms will help the breeders to develop climate-smart elite cultivars with desired characteristics to fight challenging climatic conditions.

#### 4.3.2 Crops with better nutrition

Since crops are the main source of vital nutrients like vitamins, iron, zinc, folate, fiber, limiting access to and consumption of plant-based diets can have detrimental effects on one’s health. For example, it can increase the risk of non-communicable diseases (NCD) and lead to increased nutritional deficiencies that may be challenging to ameliorate through food substitution (Scheelbeek et al., 2018). In the context of such climate change, enhancing crop nutritional quality through breeding, agronomic management, or transgenic techniques becomes essential. A promising, affordable, and sustainable method for ensuring that millions of people have healthy diets is the genetic biofortification of crops through breeding (Zenda et al., 2021).

Identification of the key genes, metabolic pathways, and QTLs that assist in understanding the genetic architecture of plant nutrient uptake is crucial for the successful improvement of crop nutritional quality. These nutritional qualities have been targeted using a variety of omics approaches, the results of which have improved GAB initiatives (Roorkiwal et al., 2021). Potential QTLs have been found in the major cereals related to nutrition such as rice (Park et al., 2019; Calayugan et al., 2020), wheat (Krishnappa et al., 2017; Ruan et al., 2020), and maize (Yang et al., 2016).

In recent work, GWAS was used to conduct comparative genomics to identify the genomic regions controlling the nutritional content of grains. In a recent GWAS study, 190 genotypes of *Eleusine coracana* were used to discover the genetic regions affecting grain nutritional content (Fe, Zn, Mg, Ca, Na, K, and proteins) (Puranik et al., 2020). Enhancing nutrient bioavailability requires a thorough understanding of the mechanisms behind crop nutrient absorption, transport, and assimilation into seeds because numerous genes and complex metabolic pathways are involved. Using omics techniques, it is feasible to comprehend the genes and metabolic pathways involved in nutrient uptake or biosynthesis, absorption, transport, assimilation, and storage (Zenda et al., 2021). For instance, a metabolic technique has been carried out to target carotenoid biosynthesis pathways and maximize the level of b-carotene in crops (rice, maize, and potato) considering that beta carotene and carotenoids are the primary precursors to vitamin A (Qutub et al., 2021; Sharma et al., 2021).

Since aflatoxin contamination has serious effects on human and animal health (Ojiewo et al., 2020), thus transcriptomics and metabolomic investigations have been performed to elucidate the aflatoxin biosynthesis in peanuts to develop the resistant varieties. The high oleic acid level is a pivotal quality attribute determining peanut flavor, stability, shelf life, and nutritional value. Therefore, high oleic acid and low linoleic acid in peanut cultivars have been developed using a various genetic technique, including QTL analysis, molecular markers, and gene editing (Amoah et al., 2020; Ojiewo et al., 2020). Therefore, these genomic advances (genomics, transcriptomics, proteomics, metabolomics, and gene editing) have made it possible to incorporate multiple agronomic traits into a single cultivar. Future developments in integrated genomic approaches are anticipated to make it easier to detect important genetic variations, identify key genes driving priority traits (high yield, stress resistance, and high-level micronutrients and proteins), and introduce those priority traits into elite cultivars, boosting economic and the nutritional value of those cultivars.

## 5 Conclusion and perspective

Recent developments in sequencing and phenotyping technologies have made it possible to extract distinctive genetic variations from the diverse spectrum of germplasm for use in plant breeding. To demonstrate how various omics techniques have anchored agricultural research and development projects, we have described several pertinent instances in this context. The identification of candidate genes, proteins, and metabolites encompassing numerous quantitative and quality traits of agronomic significance in major crops have been made possible primarily using these omics’ techniques, especially genomics, transcriptomics, proteomics, and metabolomics to study how plants respond to different biotic and abiotic stresses. Additionally, machine learning contributed to integrating the GWAS to produce a full set of data on genetic variation to predict the phenotypic parameters influencing the improvement of quality, quantity, or stress tolerance. Moreover, GWAS at the epigenome, transcriptome, protein, and metabolic levels could help the scientists to identify the detrimental and beneficial alleles for crop breeding. Through molecular breeding, or genetic engineering techniques, several reported candidate genes and metabolic pathways have been implemented in breeding programs. Integrated omics platforms are the latest avenue with significant potential in crop breeding to bridge the gap between environmental challenges and food security. Researchers will be able to implement innovative methods in forward/reverse genetics and breeding programs using an integrative omics platform that provides access to entire bioinformatics data. In conclusion, omics-based breeding research and cutting-edge technologies significantly impact crop yield, nutritional value enhancement, and stress tolerance to feed the globe. Multidisciplinary partnerships between plant scientists, computational biologists, breeding corporates, and farmers should be established to share knowledge and build a community to discover novel and ground-breaking insights in crop breeding using multi-omics techniques.

## Author contributions

**CQ, KL:** Conceptualization, Methodology. **UM, XL, YF:** Investigation, Writing - original draft. **WC, YN and JL:** Investigation. **UM, CQ, KL:** Visualization, Writing - reviewing and editing. All authors contributed to the article and approved the submitted version.
